# Pediatric Patients Discharged from the Emergency Department with Abnormal Vital Signs

**DOI:** 10.5811/westjem.2017.5.33000

**Published:** 2017-07-19

**Authors:** Josephine Winter, Michael J. Waxman, George Waterman, Ashar Ata, Adam Frisch, Kevin P. Collins, Christopher King

**Affiliations:** Albany Medical College, Department of Emergency Medicine, Albany, New York

## Abstract

**Introduction:**

Children often present to the emergency department (ED) with minor conditions such as fever and have persistently abnormal vital signs. We hypothesized that a significant portion of children discharged from the ED would have abnormal vital signs and that those discharged with abnormal vital signs would experience very few adverse events.

**Methods:**

We performed a retrospective chart review encompassing a 44-month period of all pediatric patients (aged two months to 17 years) who were discharged from the ED with an abnormal pulse rate, respiratory rate, temperature, or oxygen saturation. We used a local quality assurance database to identify pre-defined adverse events after discharge in this population. Our primary aim was to determine the proportion of children discharged with abnormal vital signs and the frequency and nature of adverse events. Additionally, we performed a sub-analysis comparing the rate of adverse events in children discharged with normal vs. abnormal vital signs, as well as a standardized review of the nature of each adverse event.

**Results:**

Of 33,185 children discharged during the study period, 5,540 (17%) of these patients had at least one abnormal vital sign. There were 24/5,540 (0.43%) adverse events in the children with at least one abnormal vital sign vs. 47/27,645 (0.17%) adverse events in the children with normal vital signs [relative risk = 2.5 (95% confidence interval, 1.6 to 2.4)].However, upon review of each adverse event we found only one case that was related to the index visit, was potentially preventable by a 23-hour hospital observation, and caused permanent disability.

**Conclusion:**

In our study population, 17% of the children were discharged with at least one abnormal vital sign, and there were very few adverse (0.43%) events associated with this practice. Heart rate was the most common abnormal vital sign leading to an adverse event. Severe adverse events that were potentially related to the abnormal vital sign(s) were exceedingly rare. Additional research is needed in broader populations to better determine the rate of adverse events and possible methods of avoiding them.

## INTRODUCTION

Fever, tachycardia, and tachypnea are frequently seen in pediatric emergency department (ED) patients.^1^,^2^ Experience suggests that children presumed to have a minor illness are often discharged from the ED despite having one or more abnormal vital signs and that they generally do not experience an adverse outcome.

Using vital signs for risk stratification has been postulated as one potential mechanism for identifying children at high risk for sepsis. Several studies have been published evaluating the diagnostic and predictive utility of vital sign abnormalities at the time of presentation and during an ED visit in pediatric patients.^2^–^5^ Additionally, several pediatric clinical prediction rules have included vital signs in their analysis of the likelihood of sepsis in febrile children.^6^–^18^ However, we are unaware of any literature examining the practice of discharging children from the ED who have abnormal vital signs at the time of discharge.

The aim of this study was to answer two questions: (1) What proportion of children discharged from the ED had at least one abnormal vital sign at the time of discharge during the study period; and (2) How often do these patients experience a significant adverse event that was likely related to the abnormal vital sign(s)?

## METHODS

We conducted a retrospective chart review over a 44-month period (April 2010 to November 2013) of all children aged two months to 17 years discharged from a large academic medical center ED. Institutional review board approval was granted.

We obtained data from two sources. First, the ED electronic health record system was queried for all children aged two months to 17 years discharged from the ED during the study period. Discharge vital signs were extracted from the patient’s medical record as their last set of vital signs taken on each patient. We defined abnormal vital signs as temperature greater than 100.4 F (38.0 C) and oxygen saturation less than 95%, while heart rate and respiratory rate were considered abnormal if outside standard published age-specific ranges.^19^,^20^

Second, our ED quality control database was reviewed for the same time period. This database has all 72-hour returns, patient complaints, internal and external referrals for morbidity and mortality review, and deaths. As the only pediatric referral center in northeastern New York State, we assumed that our quality assurance database estimates the total number of adverse events in children discharged from our ED.

Before collecting data, our research team – consisting of pediatric emergency medicine (PEM), dual-boarded emergency medicine (EM)/PEM, and EM-trained physicians – deliberated and reached a consensus on what constituted an adverse event: re-presentation to hospital and admission for ≥ five days, cardiopulmonary resuscitation, endotracheal intubation, and unexpected surgery. Patient death related to the initial visit was also included on a case-by-case basis, even if it did not take place within the 72 hours after ED discharge. Because there is no evidence-based consensus on what length of stay for a readmission constitutes an adverse event, our research team chose a longer length of stay for readmissions (five days as opposed to three days) in order to identify cases that would be very important for an emergency physician to avoid.

To further adjudicate each case, the records for all patients found to have an adverse event were independently reviewed by two study authors (one boarded in EM and another dual-boarded in EM/PEM) to determine whether (a) the adverse event could reasonably have been considered as potentially related to the initial visit; (b) the adverse event would likely have been prevented if the patient had been observed in the hospital rather than discharged; and/or (c) the adverse event resulted in death or likely permanent disability. On two occasions there was a discrepancy between the two reviewers, and a third investigator (board certified in EM) independently reviewed the case to break the tie. To minimize the rate of missed adverse events, the adjudicators were asked to categorize cases that were not clear as “having an adverse event.”

We determined the proportion of pediatric patients discharged with abnormal vital signs and the rate of occurrence of adverse events. The relative risk was calculated by comparing the rate of adverse events in children with at least one abnormal vital sign at the time of discharge vs. children discharged with normal vital signs. For each individual vital sign, we created ROC curves and calculated the area under the curve. Data analysis was performed using STATA 14.0 (StataCorp LLC, College Station, Texas).

## RESULTS

**Figure 1:**
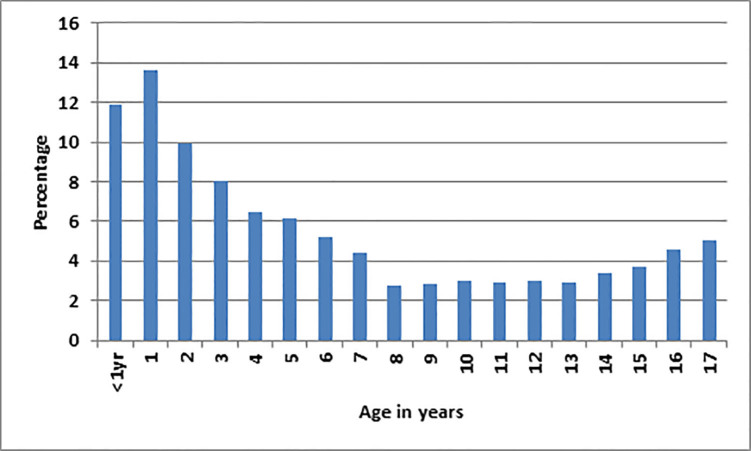
Age distribution of the 33,185 pediatric patients in a study examining the relationship between adverse outcomes and discharge from the emergency department with abnormal vital signs.

**Figure 2:**
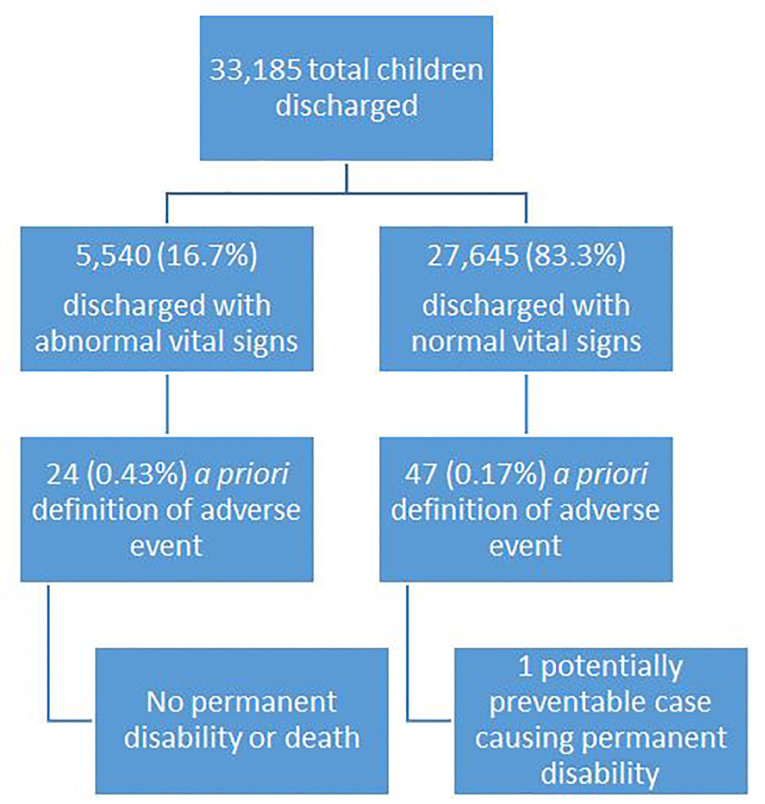
Flow diagram of discharged patients with (1) normal vs. abnormal vital signs, (2) a priori-defined adverse outcomes, and (3) preventable adverse outcomes leading to disability or death after review.

A total of 33,185 children aged two months to 17 years were discharged from the ED during the study period. Age distribution is shown in Figure 1, and additional demographics are shown in Table. Of these patients, 5,540 (17%) were discharged with at least one abnormal vital sign. A flow diagram of discharged patients with (1) normal vs. abnormal vital signs, (2) *a priori* defined adverse outcomes, and (3) preventable adverse outcomes leading to disability or death after review is presented in Figure 2.

Of the 5,540 children discharged with one or more abnormal vital signs, 24 (0.43%) met our *a priori* criteria for an adverse event (see below for categorization of outcome). Of the 27,645 patients discharged with normal vital signs, 47 (0.17%) met *a priori* criteria for an adverse event. The relative risk (RR) of *a priori* defined adverse events in patients discharged with one or more abnormal vital signs compared with those with normal vital signs was 2.5 (95%, CI [1.6 – 4.2]) and the number needed to harm (NNH) was 380 (95%, CI [252 – 767]).

Among the 24 children discharged with one or more abnormal vital signs and who had an adverse event, seven (29%) were discharged with an elevated temperature ranging from 100.5 F to 103.2 F, seven (29%) were discharged with a low oxygen saturation ranging from 92% to 94%, 16 (67%) were discharged with an age-specific abnormal heart rate, and four (17%) were discharged with an age-specific abnormal respiratory rate. Among the 5,516 children discharged with abnormal vital signs and no adverse events, there were 2,498 (45.3%) children discharged with elevated temperatures ranging from 100.4 F to 105.6, 819 (14.9%) children discharged with low oxygen saturations ranging from 66% to 94%, 3,092 (56.1%) children discharged with an age-specific abnormal heart rate, and 483 (8.8%) children discharged with an age-specific abnormal respiratory rate. When creating ROC curves for each of the vital signs individually, we found that pulse, respiration, temperature, and oxygen saturation had poor discrimination for predicting adverse events (area under the curve 0.57, 0.54, 0.45, 0.59, respectively). See supplemental material for ROC curves.

When each adverse event was adjudicated, it was found in the abnormal vital signs group that five patients required surgery (none of which sustained permanent morbidity from a complication secondary to delayed presentation), 17 patients were admitted to the hospital for five days or longer (none with likely permanent morbidity/disability), and two patients died. On review of the deaths, one was judged to be unrelated to the index visit (unrelated accidental injury), and the other death was due to infection and not believed to be potentially preventable by hospital observation. Among the 17 patients admitted to the hospital for five days or longer, 12 were admitted primarily because of infectious related problem, three were admitted primarily because of gastroenterological or metabolic condition, and two were admitted primarily because of an exacerbation of a chronic condition.

In the normal vital signs group, 11 patients required surgery (one of whom sustained permanent morbidity from a complication secondary to delayed presentation), 36 patients were admitted to the hospital for five days or longer, and no patients died. Among the 36 patients admitted to the hospital for five days or longer, 17 were admitted primarily because of infectious related problem, five were admitted primarily because of gastroenterological or metabolic condition, and 14 were admitted primarily because of rheumatologic, cardiac, otolaryngological, hematology/oncology, or other problem related to a chronic disease.

In summary, after further manual review of each adverse event, one case (in the normal vitals group) was deemed by the reviewers to have been related to the index visit, potentially preventable, and to have led to permanent disability. This patient was a pre-school aged child who presented during the index visit with intermittent abdominal pain and a normal testicular exam documented in the record, who subsequently represented with a testicular torsion requiring orchiectomy. The case was judged to be potentially preventable with a hospital admission and to have led to permanent disability by two out of the three adjudicators.

## DISCUSSION

In our study population, it was relatively common for pediatric patients to be discharged from the ED with abnormal vital signs, and it was rare for these patients to experience adverse events.

The rate of adverse events was greater in children discharged with abnormal vitals than those discharged with normal vitals (RR = 2.5, 95%, CI [1.6 – 4.2]). This is not surprising since vital signs are usually considered to have at least some utility in predicting whether a child is sick. Nevertheless, it is important to contextualize the statistically significant relative risk as the rates of adverse events in both cohorts (children discharged with normal vitals and children discharged with abnormal vitals) were very low.

Furthermore, it was important to us to not only determine the rate of our *a priori* defined adverse events, but to also assess the nature and severity of each adverse event. For example, if a child with bronchiolitis is discharged from the ED and subsequently requires hospital admission for several days but suffers no permanent morbidity, this may be considered a typical progression of the illness rather than a severe adverse event. Conversely, a child who appeared well enough to discharge home but who returned with meningitis and permanent brain injury would be exactly the kind of disastrous case that we would most want to identify. When each case was reviewed for whether there was an event that was preventable and/or caused permanent disability or death, there were so few cases (one case of potentially preventable permanent disability and no potentially preventable deaths) that any type of comparison between the abnormal and normal vital signs groups would not be meaningful.

## LIMITATIONS

Our study has several limitations. First, while our data represents 33,185 discharges, there were very few adverse events, deaths, and/or cases of permanent disability in our single-site retrospective study. Given the relatively small number of adverse events, we were only able to use a single “cutoff” value for each abnormal vital sign in our data analysis. Further studies, using larger datasets with greater numbers of serious adverse events, would be needed to determine vital sign thresholds or collections of abnormal vital signs that predict unsafe discharges.

Second, we assumed that our own morbidity/mortality review process collected all major adverse events in discharged patients. While we believe that this methodology was acceptable for this particular study, the study would have been strengthened if it had been linked with statewide registries and/or death records to ensure that there were no additional significant adverse events of which we were not aware. Third, categorizing adverse events is often subjective. Because we wished to identify more serious adverse events – cases in which it would be highly important for an emergency physician to take great pains to avoid – we defined a longer inpatient stay of five days (as opposed to three days) to be an adverse event. We also tried to mitigate this subjectivity as we best we could by basing our primary analysis on a set of predefined criteria, and then by adjudicating each case to see if it was preventable and/or caused permanent disability or death.

Fourth, we do not know the route by which temperature was taken since this information is often not recorded in our electronic heath record system. However, we believe that our study represents a real-life scenario, since the research data consists of the last set of vital signs that the emergency provider saw before discharging the patient. Finally, because we specifically looked at patients who were already discharged, we do not know how many patients may have been admitted to the hospital solely because they had one or more abnormal vital signs at the time of planned discharge on the index visit, and hence would not have been included in our analysis. We acknowledge that vital signs are only a piece of the clinical puzzle, and mature emergency providers must take into account the entire clinical picture, including clinical appearance, social situation, potential for follow-up, etc.

## CONCLUSION

In this retrospective review at one institution, 17% of pediatric patients were discharged from the ED with one or more abnormal age-specific vital signs. Heart rate (66%) was the most common abnormal vital sign leading to adverse event. Adverse events were 2.5 times more common (95%, CI [1.6 – 4.2]) in patients discharged with abnormal vital signs compared to those discharged with normal vital signs, but the frequency of adverse events in both groups was low (0.43% in the abnormal vitals group and 0.17% in the normal vitals group). Furthermore, after reviewing each adverse event, there was only one case that led to permanent disability and may have been preventable if the patient had been observed or admitted rather than discharged. Further study in broader patient populations is needed to verify our results, and identify characteristics of ED discharge vital signs that may be useful to guide patient care.

## Supplementary Information



## Figures and Tables

**Table:** Demographic features of the study population.

	Percentage
Age	
2 months to 1 year	11.9%
1 year to 4 years	37.7%
5 years to 10 years	24.5%
11 years to 17 years	25.6%
Gender	
Female	46.2%
Male	53. 8%
Race/ethnicity	
White	56.2%
Black	28%
Other	15.8%
Identified as Hispanic	10.8%
Insurance status	
Insured	95.7%
Uninsured	4.3%

* Patients having Medicaid or Medicaid managed care were considered insured.
