# Supplementary material for: Pediatric Patients Discharged from the Emergency Department with Abnormal Vital Signs
Source: West J Emerg Med. 2017 Jul 19;18(5):878–83. doi: 10.5811/westjem.2017.5.33000 (PMC5576624; doi:10.5811/westjem.2017.5.33000)
Supplement: Supplementary file 1 [file wjem-18-878-s001.docx]

**Supplemental Figure.** ROC Curves for pulse, temperature, respiratory rate, and oxygen saturation to predict an adverse event.

Pulse

Temperature

Respiratory rate

Oxygen saturation
